# Hijacking QSK1: How pathogens turn a plant defense guardian into an accomplice

**DOI:** 10.1093/plcell/koae262

**Published:** 2024-09-25

**Authors:** Shanice S Webster

**Affiliations:** Assistant Features Editor, *The Plant Cell*, American Society of Plant Biologists; Howard Hughes Medical Institute, Chevy Chase, MD 20815, USA; Duke University Department of Biology, Durham, NC 27708, USA

Plants and pathogens are in constant warfare. During infection, pathogens deploy virulence effectors to manipulate and suppress host defenses. Many immune receptor kinases are well-characterized; however, the large repertoire and multifunctionality of pathogen effectors would leave room for novel immune receptor kinases and their mechanisms to be discovered. Yukihisa Goto, Yasuhiro Kadota, and colleagues identify QSK1 as a novel component of the pattern recognition receptor kinase complex in Arabidopsis (*A. thaliana*) and reveal new insights into how the pathogen effector HopF2 exploits QSK1 to desensitize plants to pathogen attack (Goto et al. 2024).

A hallmark of plant defense is the recognition of microbial molecules by receptor kinases, which then associate and activate co-receptors. For example, FLS2 and EFR are receptor kinases that bind bacterial flagellin and elongation factor, respectively. This binding leads to the formation of a receptor kinase complex, which activates downstream signaling pathways, resulting in ROS production, Ca^2+^ influx, and mitogen-activated protein kinase (MAPK) activation, all of which help to suppress pathogen growth. To overcome this immune response, pathogens use virulence effectors to either degrade or target receptor kinase activity. Using comparative immunoprecipitation analysis combined with mass spectrometry of FLS2, EFR, and RBOHD complexes, Goto et al. identify the receptor kinase QIAN SHOU KINASE1 (QSK1) as a component of the receptor kinase complex. The authors show that interaction between QSK1 and the receptor kinases is ligand independent and is stable with and without flagellin peptide elicitation, suggesting that QSK1 is likely an essential part of the receptor kinase complex (Fig. 1A).

**Figure 1.**
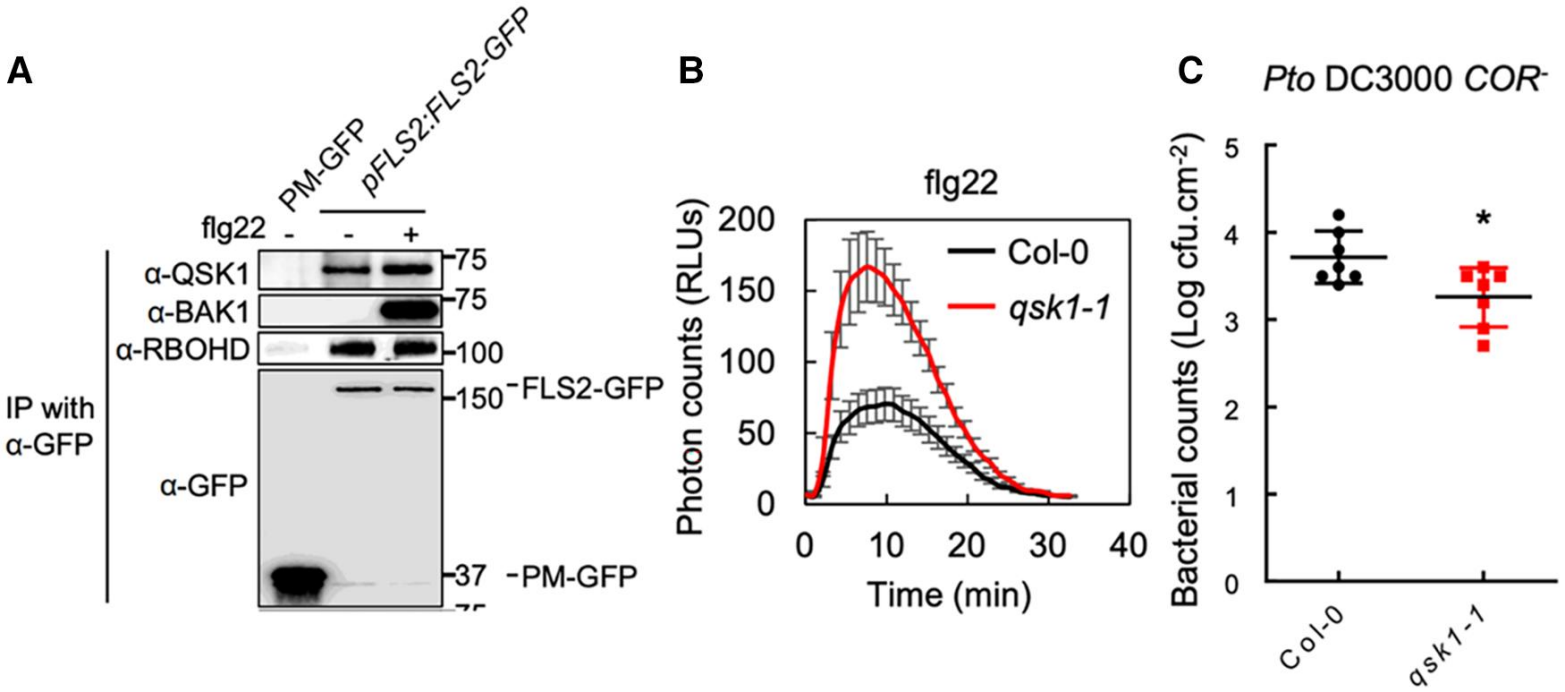
QSK1 function in plant immunity. **A)** QSK1 associates with FLS2 in *A. thaliana* plants independent of flagellin peptide elicitation. **B)**  *A. thaliana qsk1* null mutant shows increased ROS production compared with wild-type Col-0 plants, indicating increased immune response. **C)**  *qsk1* mutant shows increases resistance to the plant pathogen *P. syringae* lacking the coronatine toxin. Adapted from Goto et al. (2024), Figures 2, 3.

To investigate the impact of QSK1 on immune signaling in plants, the authors examined an Arabidopsis *qsk1* null mutant. They show that the *qsk1* mutant increases ROS production and MAPK activation and has greater resistance to an attenuated version of the pathogenic strain *Pseudomonas syringae tomato* (*Pst*) DC3000 (Fig. 1B and 1C). In contrast, plants with higher levels of QSK1 expression showed reduced immune signaling and increased susceptibility to the pathogen. These results indicate that QSK1 negatively regulates immune signaling and consequently increases susceptibility to pathogens.

The authors hypothesized that QSK1 might impact the protein stability of its receptor kinase partners to suppress their function. Only FLS2 protein levels were altered in the *qsk1* plant mutant compared with the wild type, and this modulation of FLS2 protein accumulation was independent of its catalytic domain. Furthermore, the authors observed a decrease in plasma membrane localization of FLS2 in the QSK1 overexpression lines, indicating that QSK1 interacts with FLS2 and negatively regulates its accumulation.

Receptor kinases are critical targets for pathogen effectors like HopF2 from *P. syringae Pst*. Given QSK1's role in suppressing immunity, pathogens could exploit QSK1 to reduce FLS2 levels and shut down immunity. Indeed, Goto et al. demonstrate that QSK1 interacts with HopF2 and is required for HopF2 stability. Notably, using a transgenic plant line where HopF2 expression levels can be controlled, the authors show that FLS2 protein levels and localization were strikingly reduced following induction. This highlights QSK1 as a regulator of HopF2 stability and its role in inhibiting plant immunity.

Interestingly, HopF2 also further reduces receptor kinase levels through a transcription-dependent mechanism. The authors use transcriptomic studies to monitor gene expression of wild-type and transgenic HopF2 overexpression lines. They found that HopF2 reduces transcription of several receptor kinases including *fls2*. Together, these findings demonstrate that HopF2 functions by reducing the levels of receptor kinases at the plasma membrane, thereby diminishing the plant's ability to sense pathogens and induce the appropriate immune responses.

QSK1 is reported to have functions in drought stress (Chen et al. 2021) and lateral root formation (Wang et al. 2022). This work highlights yet another intriguing function of QSK1. Endogenous QSK1 is key to maintaining steady-state levels of receptor kinases and likely also functions to avoid unnecessary and excessive immune responses. These discoveries not only deepen our understanding of QSK1's multifaceted roles in plant biology but could pave the way for developing new strategies to enhance crop resilience against pathogens.

## Data Availability

No new data were generated or analysed in support of this research.

## Dive Curated Terms

The following phenotypic, genotypic, and functional terms are of significance to the work described in this paper:

FLS2 Gramene: AT5G46330
FLS2 Araport: AT5G46330

EFR Gramene: AT5G20480
EFR Araport: AT5G20480

RBOHD Gramene: At5g47910
RBOHD Araport: At5g47910

QSK1 Gramene: AT3G02880
QSK1 Araport: AT3G02880
